# Numerical simulation on seismic pounding damage in a simply-supported steel bridge

**DOI:** 10.1016/j.heliyon.2023.e22297

**Published:** 2023-11-15

**Authors:** Fan Shi, Dongsheng Wang, Lei Tong, Weijian Tang

**Affiliations:** School of Civil and Transportation Engineering, Hebei University of Technology, Tianjin 300401, China

**Keywords:** Steel bridge, Pounding damage, Numerical simulation, Preventive measure, Identification method

## Abstract

Steel bridges are generally considered to perform well during seismic activity. Nevertheless, they still suffered much unexpected seismic damage in the Kumamoto earthquake, especially seismic pounding damage. Previous studies on bridge pounding damage have generally focused on reinforced concrete bridges. However, steel bridges' dynamic characteristics are more complex, the stiffness of each component varies significantly, and relevant research remains limited. Therefore, the numerical simulation method is adopted in this paper to study the pounding damage of simple-supported steel bridges under seismic events in detail. The multiscale, fine three-dimensional finite element model was built using the general finite element calculation platform Abaqus, and dynamic implicit analysis was performed. Numerical results show that large and near-fault seismic activity results in obvious pounding damage to steel beams. Specifically, longitudinal pounding causes damage to the steel beam's ends; however, the damage is typically localized and mild. Lateral pounding further causes direct damage to the steel beams, resulting in extensive and serious damage. Horizontal pounding which combines longitudinal and lateral causes rotation of the bridge deck and aggravates the lateral damage to steel beams. In addition, a pounding identification method based only on displacement data is proposed, and a feasible preventive measure for lateral pounding damage is suggested.

## 1 Introduction

Steel is attractive in the application of bridge structures due to its good material ductility and high strength-to-weight ratio. Steel bridges have exciting potential, especially in terms of seismic performance [1]. Over the past few decades, many steel bridges have been built and experienced seismic events [2]. Housner and Thiel [3] summarized the bridge damage from the 1994 Northridge earthquake and concluded that steel bridges generally showed better seismic performance. The 1995 Kobe earthquake caused more damage to steel piers, including complete collapse and local buckling instability [4]. These early steel bridges’ seismic damage promoted the advancement of steel bridge seismic design methods. Buckle et al. [5] summarized the seismic design research of bridges with a steel-plate girder superstructure and provided design suggestions. Usami and Ge [6] proposed a performance-based approach to the seismic design of steel bridge systems. The method consists of two menus, which are displacement-based and strain-based, respectively. However, these advances do not guarantee that steel bridges will not be damaged during a seismic event.

**Fig. 1:**
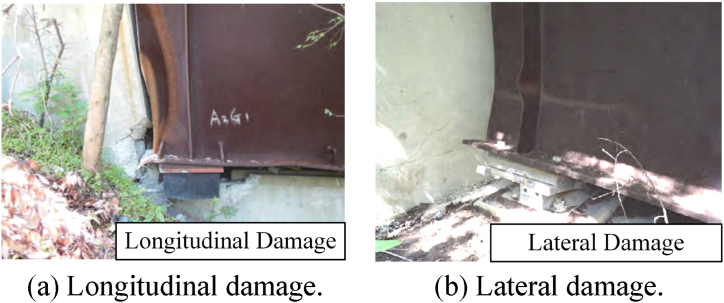
Steel beams' damage in Kumamoto earthquake [7].

Steel bridges designed according to modern seismic codes were tested in the 2016 Kumamoto earthquake. Although there was no serious collapse damage to the steel bridges [7], the seismic damage during the seismic event revealed some new characteristics. As shown in Fig. 1(a) and (b), the steel beam's damage is mainly reflected in the longitudinal pounding and lateral buckling damage. In addition, buckling and disconnection of stiffeners and lateral braces appeared. Mya Nan et al. [8] investigated the severely damaged Tawarayama Bridge, particularly the buckling of the lower lateral members, suggesting that the buckling design of the lower lateral members should be considered. Gibe et al. [9] discussed the failure process and limit state of steel bearings in the Kumamoto earthquake, especially under loads in the vertical bridge axis. Mustafa and Miki [10] examined the performance of the side blocks in an elevated girder bridge system and proposed a retrofit plan. In general, the focus of the study on steel bridge damage in the Kumamoto earthquake began to lean toward steel beams' lateral damage. Further, the damage may be exacerbated due to the pounding of steel beams caused by seismic. The apparent seismic performance of steel structures may have given the steel bridge a “foolproof” veil, while sound design and detailing practices are still required for steel bridges to achieve acceptable seismic behavior.

Scholars have studied the pounding damage of traditional reinforced concrete bridges resulting from seismic. Jankowski et al. [11] studied the pounding between adjacent superstructure segments in elevated bridges and suggested effectively improving bridge performance by placing hard rubber bumpers between segments. Zhu et al. [12] used a three-dimensional nonlinear beam model to evaluate the impact countermeasures and serviceability of elevated bridges with longitudinal limiters and buffers during seismic excitation. Chouw and Hao [13] explored the effects of ground-motion spatial variation and soil–structure interaction on the pounding response of two adjacent bridge frames. Bi et al. [14] investigated the minimum total gap that a modular expansion joint must have to avoid pounding at the abutments and between bridge decks. The effects of ground-motion spatial variations, dynamic characteristics and the depth, and stiffness of local soil on the required separation distance were also analyzed. Dimitrakopoulos [15] revisited the seismic response of short skew bridges and proposed a novel nonsmooth rigid body approach to study the seismic pounding response involving oblique friction multicontact phenomena. Banerjee et al. [16] used nonsmooth techniques to consider deck-abutment pounding interactions of curved bridges under deck rotation. These studies reveal the law of bridge pounding response from different angles; however, they all simplify the bridge to a spring-mass model or a beam bar model. The pounding force is borne evenly by the entire beam section in these simplified models, thus it is difficult to discuss the range and extent of the beams’ damage.

Bi and Hao [17] used the finite element code LS-DYNA to analyze the pounding response of a bridge under spatially varying ground motion. The study used a three-dimensional finite element model to capture the eccentric poundings of the bridge deck and suggested that these poundings may induce local damage at the bridge deck's corners. In addition, Hao et al. [18] reviewed the seismic pounding response of bridges, including impact models, numerical simulations, experimental investigations, and pounding mitigation methods. Sha et al. [19] studied the pounding responses of an isolated continuous girder bridge under longitudinal non-pulsed and pulsed ground motions based on the multiscale simulation scheme. Li et al. [20] experimentally studied the adjacent pounding effect of a midspan curved bridge with a longitudinal slope and analyzed the influence of connection parameters, such as gap size and longitudinal slope. Poundings occur not only between beams and abutments under longitudinal ground motions but also between beams and shear keys under transverse ground motions. Xu et al. [21] proposed a new type of shear key for the retrofit of existing highway bridges and experimentally studied its seismic behavior under repeated monotonic loading. Meng et al. [22] studied the effects of shear keys and CRTS-II track systems on the seismic behavior of single-supported bridges of the high-speed rail under transverse seismic excitation. In general, the seismic pounding response of reinforced concrete bridges has been studied in depth. Simple spring-mass models, engineered beam bar models, and detailed three-dimensional models have been developed.

However, the study of the seismic-induced pounding of steel bridges remains superficial. DesRoches et al. [23], for example, used fiber models to study the seismic response of multispan steel bridges in the central and southeastern United States and concluded that fixed steel bearings are the most vulnerable component during seismic pounding events. The use of lead-rubber bearings is the most effective retrofit measure for reducing seismic vulnerability of steel bridges; it should be noted, however, that the increased flexibility may result in pounding between steel bridge decks [24]. Won et al. [25] studied the seismic-induced pounding effect on bridge piers by analyzing the dynamic response of a three-span simply supported steel girder bridge. The response of the piers is evaluated by an idealized analytical model that reflects the random characteristics of seismic excitations. Zheng et al. [26] evaluated the pounding interaction between bridge abutments and steel beams under nonuniform seismic excitation. A detailed nonlinear finite element model was established, and the pounding element was simulated by a trilinear compression gap. These studies have a limited understanding of pounding damage in steel bridges; as such, it is necessary to further examine seismic damage to steel bridges, as shown in Fig. 1.

Although studies have discussed seismic-induced pounding damage to bridges, challenges remain for steel bridges, given the damage resulting from the Kumamoto earthquake: 1) the cross-sectional area of steel beams is small, and local stiffness is low (compared with traditional concrete beams), so the pounding load may cause local deformation damage, even buckling failure; 2) the transverse stiffness of the steel beam is low and uneven, damage to a steel beam under lateral seismic pounding may be serious; 3) the scope of pounding damage to steel beams and the influence of different damage positions on the overall force performance are not clear; 4) refined numerical simulation methods must be adopted due to the mechanical nonlinearity of steel and contact behaviors. Therefore, a further refined three-dimensional numerical simulation study of seismic-induced pounding damage of steel bridges is necessary. In general, the research aim of this study is to clarify the seismic pounding damage of simply-supported steel bridges. Specifically, a representative simply-supported steel bridge is analyzed by numerical simulation method to illustrate the seismic pounding damage characteristics of this type of bridge.

This paper is organized as follows. Background and motivations are introduced in Section 1. The modeling method of the steel bridge numerical model is illustrated in Section 2. Ground motions that need to be inputted are introduced and processed in Section 3. Section 4 discusses in detail the numerical simulation results and preventive measures for pounding damage in steel bridges. Finally, in Section 5, the main conclusions are drawn. The identification method for pounding events is introduced in Appendix A.

## 2 Bridge model

### 2.1 Structural details

Steel bridges have many structural forms, among which the most commonly used in engineering are slab-on-girder steel bridges. They are generally composed of concrete decks and steel I-beams, so they can also be called steel–concrete composite girder bridges. The bridge model in this paper is based on a built simply-supported steel–concrete composite girder bridge. This bridge is located on the Yanqing-Chongli Highway in China, which was completed in 2020 and served the traffic needs of the Olympic Winter Games Beijing 2022. The design of the selected bridge is basically consistent with the design in the General Diagram of the Superstructure of the Highway Steel-Concrete I-Beam (Simply Supported Part). The standard design scheme of this type of bridge is given in the General Diagram, which is an important reference for engineers when designing bridges. In addition, the selected bridge is similar to those that exhibited typical earthquake damage, such as having high-stiffness abutments and steel beams exceeding the length of the concrete deck. Based on the above reasons, the simply-supported steel bridge selected is considered representative of this bridge type. Therefore, studying the seismic pounding damage of this bridge is of great significance to understanding the seismic damage characteristics of this type of bridge.

**Fig. 2:**
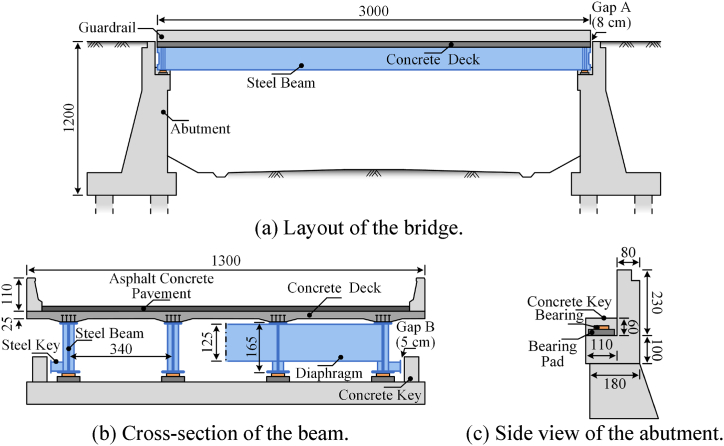
Layout and dimensions of the bridge (unit: cm).

Fig. 2 shows the layout and dimensions of this one-span bridge. As shown in Fig. 2(a), the superstructure is simple-supported steel–concrete I-beams (span of 30 m). The substructure is made of U-shaped abutments and pile foundations. Gaps of 80 mm were introduced between the abutments and the bridges to avoid binding forces on the girders due to longitudinal deformations caused by temperature fluctuations, traffic loads, etc. This type of gap is denoted as Gap A in Fig. 2.

The total width of the bridge is 12.75 m, including four I-beams. The spacing between each beam is 3.4 m, and the beam height is 1.65 m. The cross-section of the bridge beam is shown in Fig. 2(b). The steel beams are welded from the top, bottom, and web plates, which are 25, 30, and 13 mm thick. The transverse connection between the steel beams is reinforced by steel diaphragms. At the end of steel beams, steel diaphragms are wrapped in concrete diaphragms. The bridge deck is made of reinforced concrete, the thickness of the standard section is 25 cm, and the steel beam support area is thickened to 35 cm. To prevent the bridge from falling beam failure, steel shear keys are provided at both ends of the side beams, and concrete shear keys are set at both ends of the abutment. Gaps of 50 mm were introduced between the steel and concrete shear keys; this type of gap is denoted as Gap B in Fig. 2. Eight road bridge basin bearings are set on the two abutments to support steel beams. They are two unidirectional movable bearings and six bidirectional movable bearings, each with a movement of ±100 mm. The detailed dimensions of the abutment are shown in Fig. 2(c).

### 2.2 Element and material properties

A finite element model (FEM) is built using the general finite element calculation platform Abaqus [27] and solved by dynamic implicit analysis. To accurately simulate the seismic pounding behavior of steel beams, a small mesh size is required, which significantly increases computational costs. Calculation accuracy and cost can be better balanced, considering that the pounding behavior is concentrated on the steel beam end. The bridge model is modeled using the multiscale fine modeling method, and different components are divided into meshes with different sizes.

**Fig. 3:**
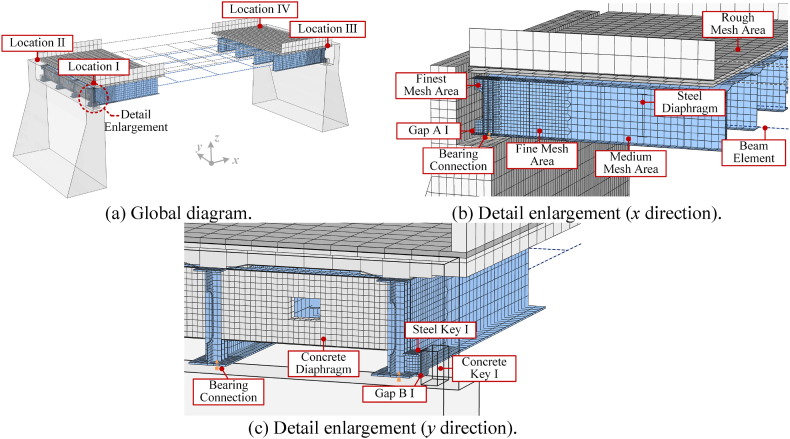
3D multiscale FEM of the bridge.

An S4R plate element is used for the two ends of the steel beam, which may be unevenly deformed, torsional, and buckled during an earthquake, while the B31 beam element is used for the middle part of the steel beam. The connection at the interface should ensure that no additional constraints on the plate model are added without losing the freedom of the beam model. Thus, at the interface connection of the plate and beam element, the transmission of translational and rotational freedom is handled according to the principles of displacement coordination and flat section, respectively. To ensure uniformity of stiffness between different element scales in the model, the beam model needs to be refined according to the space grillage method. The basic idea of this method is to replace the actual bridge superstructure with an equivalent space grillage and concentrate the bending stiffness and torsional stiffness in each lattice into the nearest grillage, so the lateral action of the bridge must be transmitted through the transverse portion of the grillage. Virtual beams were introduced into the model as transverse parts that can only transmit stiffness without weight. The application of transverse virtual beams not only takes into account the lateral stiffness of the structure but also ensures the correct self-weight load. The abutment is modeled using a C3D8R solid element. The overall FEM of the bridge modeled by the multiscale modeling method is shown in Fig. 3(a). For ease of understanding, the four corners of the bridge model are named Location Ⅰ, Location Ⅱ, Location Ⅲ, and Location Ⅳ, respectively. The components of the corresponding locations are also named Gap A Ⅰ, etc.

In addition, numerical convergence tests on multiple mesh sizes (25, 50, and 100 mm) showed that the results from 50 mm and smaller mesh were similar but computationally inexpensive. The 50 mm finest mesh is only suitable for the length range of the steel beam 0.25 m from the end. The fine mesh of 100 mm is used for the transition part of the steel beam and the diaphragms; the medium mesh of 200 mm is used for the rest of the steel beam; the rough mesh of 500 mm is used for all concrete components. Fig. 3(b) and (c) show the division scheme of the above mesh size from the *x* and *y* directions, respectively.

For the simulation of steel (Q345qE), we used a high-precision hysteresis constitutive model, considering the mixed hardening effect of steel being adopted. The constitutive model includes nonlinear isotropic and kinematic hardening. Researchers typically use this constitutive model, which has proven to be a good predictor of nonlinear mechanical responses in steel [28].

The constitutive model obeys the Mises yield criterion; the function is defined by Eq. (1) as

(1) $$F=f\left(\sigma -\alpha \right)-\sigma^{0}=0\text{,}$$

where *σ*^0^ is the yield stress and *f* is the equivalent Mises stress or Hill's potential concerning the back stress *α*.

The model assumes associated plastic flow, which is defined in Eq. (2) as

(2) $${\dot{\varepsilon }}^{p}={\dot{\bar{\varepsilon }}}^{p}\frac{\partial F}{\partial \sigma }\text{,}$$

where $\dot{\varepsilon }$ ^p^ is the rate of plastic flow and $\dot{\bar{\varepsilon }}$ ^p^ is the equivalent plastic strain rate.

The evolution of the yield surface size is a function of the equivalent plastic strain and is calculated by Eq. (3), which represents the isotropic hardening component to depict the expansion of the yield surface.

(3) $$\sigma^{0}={\sigma |}_{0}+Q_{\infty }\left(1-e^{-b{\bar{\varepsilon }}^{p}}\right)\text{,}$$

where *σ*|_0_ is the yield stress at zero plastic strain, and *Q*_∞_ and *b* are material parameters.

The kinematic hardening represents the translation of the yield surface and is calculated by Eq. (4) as

(4) $$\dot{\alpha }=C{\dot{\varepsilon }}^{p}-\gamma \alpha {\dot{\bar{\varepsilon }}}^{p}\text{,}$$

where *C* and *γ* are material parameters.

**Fig. 4:**
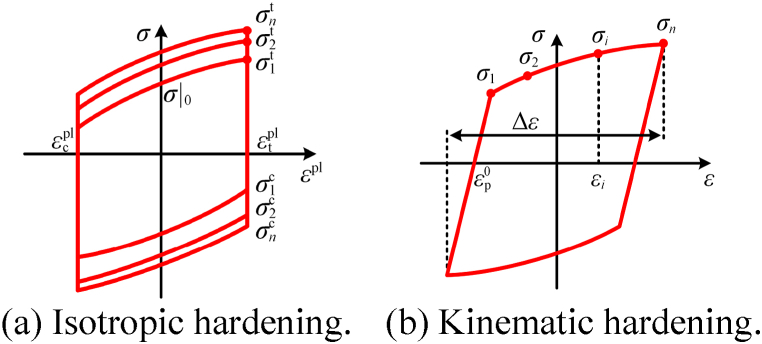
Mixed hardening constitutive model of steel.

Fig. 4(a) and (b) show the mixed hardening constitutive model; Table 1 shows the material parameter calibration results of steel. Young's modulus, density, and Poisson's ratio of the steel material are 2.06 × 10^5^ MPa, 7850 kg/m^3^, and 0.2, respectively. In this study, since the main focus is on the seismic pounding response of steel beams, only the elastic behavior of concrete materials is considered. Young's modulus, density, and Poisson's ratio of the concrete material are 3.45 × 10^4^, 2600 kg/m^3^, and 0.2, respectively.

**Table 1:** Material parameters of the constitutive model for steel Q345qE.

*σ*|_0_/MPa	*Q*_∞_/MPa	*b*_iso_	*C*_1_/MPa	*γ*_1_	*C*_2_/MPa	*γ*_2_	*C*_3_/MPa	*γ*_3_	*C*_4_/MPa	*γ*_4_
429	21	1.2	7993	175	6773	116	2854	34	1450	29

### 2.3 Contact and boundary conditions

For beam ends and shear keys that may cause seismic pounding damage, we establish the general contact relationship to achieve free contact in space between the three-dimensional point-to-surface and surface-to-surface. The general contact behavior consists of normal and tangential contact behavior. Due to the effectiveness and convergence of the penalty function method in implicit analysis, this method is adopted to establish the “normal hard contact” relationship for the contact interface. Separation after contact is allowed, and the contact stiffness is linear. Isotropic friction is considered for tangential behavior, and the friction formula uses the penalty function. The friction coefficient between the steel plate and concrete surface is 0.4 [29]. With this method, the interface contact force is transmitted between the master and slave surfaces according to the penalty stiffness and penetration conditions when contact occurs. The affected location is spatially arbitrary and not *a priori*.

**Fig. 5:**
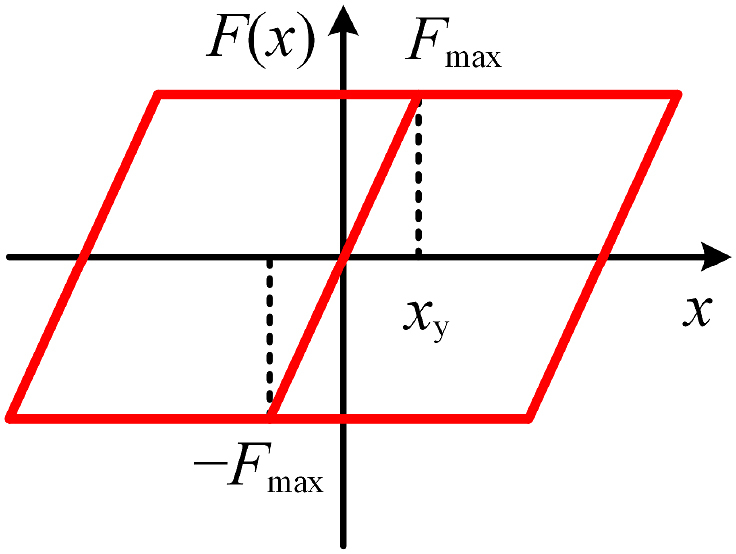
Resilience model of bearing.

The force–displacement relationship of the bearing is simulated using the elastic-damping connecting element. The upper and lower nodes of the connecting element are constrained by a multipoint (beam type) with the bearing area of the steel beam bottom and the abutment. As shown in Fig. 5, the force–displacement relationship of a movable basin bearing can be expressed as the bilinear ideal elastoplastic model.

The bearing's critical sliding friction and initial stiffness are calculated according to Eqs. (5), (6).

(5) $$F_{\max}=\mu_{d}\cdot R\text{,}$$

(6) $$k=F_{\max}/x_{y}\text{,}$$

where *F*_max_ is the critical sliding friction of the bearing (kN), *μ*_d_ is the sliding friction coefficient (generally 0.02), *R* is the gravity of the superstructure supported by the bearing (kN), *k* is the initial stiffness of the bearing (kN/m), and *x*_y_ is the yield displacement (m) of the movable basin support (generally 0.002–0.005 m).

In this study, the contact relationship between steel beams and the concrete bridge deck is simulated based on the hypothesis of tight bonding; that is, it is considered that the two do not produce relative slip and adopt the tie constraint. For concrete beams and steel beams in the model, there is a binding connection between the two parts. Since the pounding only occurs at the beam ends, the beam in the middle part always remains elastic, this simplification is reasonable. It can be further assumed that the abutments are rigidly fixed on the ground to focus on the seismic-induced pounding damage of the steel bridge, due to the small span, high stiffness, and the foundation soil with small depth. In addition, geometric nonlinearity is performed in the dynamic implicit analysis, as large deformation buckling failures may occur in steel beams locally under seismic action.

## 3 Ground motions

### 3.1 Ground-motion records

According to the ground soil conditions of the bridge site (Category B), records of representative ground-motion acceleration with far and near faults were selected as the input boundary conditions for time-history analysis. The ground motion with a far fault can be helpful for understanding the basic situation of seismic-induced pounding damage in steel bridges. Strong velocity pulses, which may exacerbate the pounding failure of steel beams, are among the most significant features of near-fault ground motions. Following Baker's suggestions [30], ground motion records from Station El Centro Array #9 in the Imperial Valley earthquake and Station TCU052 in the Chi-Chi earthquake were selected as input boundary conditions for the far and near faults, respectively. The original information of ground motion records is shown in Table 2.

**Table 2:** Original information of ground-motion records.

NO.	Earthquake	Year	Station	Magnitude	Fault distance/km
1	Imperial Valley	1940	El Centro Array #9	7.2	8.3
2	Chi-Chi	1999	TCU052	7.6	0.7

Both sets of ground motion records contain two horizontal components and one vertical component. Although the vertical component has an influence on the bridge's seismic response, it is mainly reflected in the vertical impact and uplift effect at the side spans. However, the transverse stiffness of the steel I-beam's end is significantly lower than the vertical stiffness. Under seismic action, lateral pounding damage may lead to a serious decline in the steel beam's mechanical properties. Further considering the numerical convergence and computational cost, this study focuses on the horizontal pounding damage of seismic-induced steel bridges. Therefore, it is sufficient to use two horizontal ground-motion recording components for numerical analysis. The records of the NS and WE components are used as input boundary conditions in the longitudinal (*x* direction) and transverse (*y* direction) for the bridge model, respectively.

**Fig. 6:**
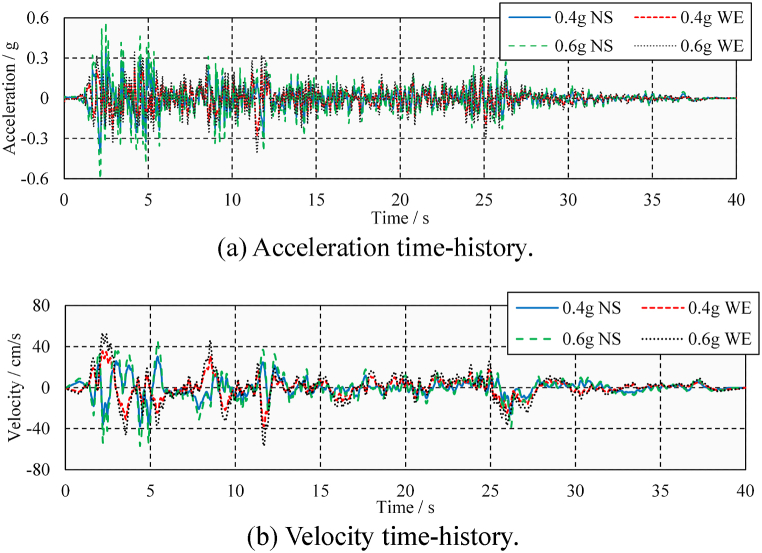
Ground-motion time-history record of El Centro Array #9 Station.

**Fig. 7:**
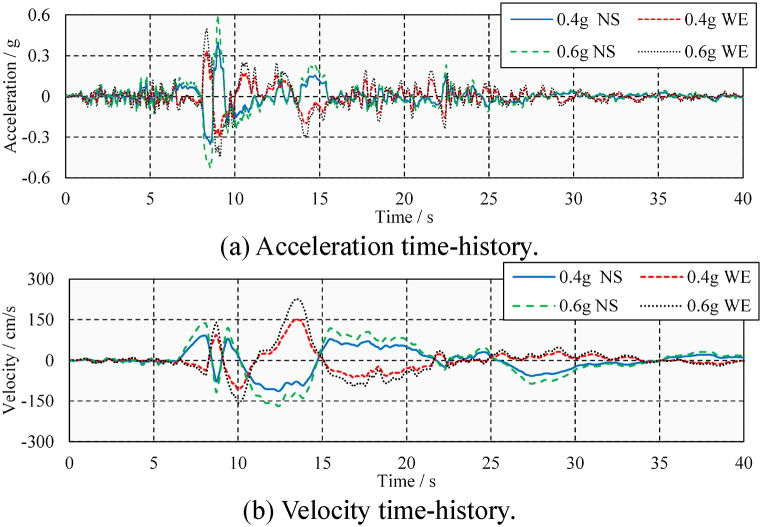
Ground motion time-history record of TCU052 Station.

In addition, the analysis of the seismic-induced pounding damage is further divided into two cases: medium and large ground-motions. The two ground motions are distinguished by the different peak ground accelerations (PGAs). Referring to the general design guidelines, the PGAs for medium and large ground-motions are considered to be 0.4 and 0.6 times the gravity acceleration, respectively (0.4 and 0.6 g). The amplitude modulation factors for ELC are 1.28 and 1.92, respectively. The amplitude modulation factors for TCU are 0.95 and 1.43, respectively. It can be seen that the ground-motions after amplitude modulation do not cause excessive distortion. Fig. 6(a) and (b) show the ground-motion time-history records of El Centro Array #9 Station (hereinafter referred to as ELC). Fig. 7(a) and (b) show the ground-motion time-history records of TCU052 Station (hereinafter referred to as TCU).

### 3.2 Response spectrums

The adoption of Rayleigh damping in the dynamic analysis is a convenient option. For reinforced concrete bridges, the acceleration response spectrum of a single-degree-of-freedom system can generally be calculated considering the damping ratio of 0.05 (*ζ* = 5 %). For steel bridges, the damping ratio is lower, generally 0.03 (*ζ* = 3 %). Damping coefficients *a*_1_ and *a*_2_ are calculated as Eq. (7) [31].

(7) $$\left\{\begin{array}{c} a_{1}\\ a_{2} \end{array}\right\}=\frac{2\zeta }{\omega_{1}+\omega_{2}}\left\{\begin{array}{c} \omega_{1}\omega_{2}\\ 1 \end{array}\right\}\text{,}$$

where *ω*_1_ and *ω*_2_ are the structure's first and second natural frequencies, respectively.

**Fig. 8:**
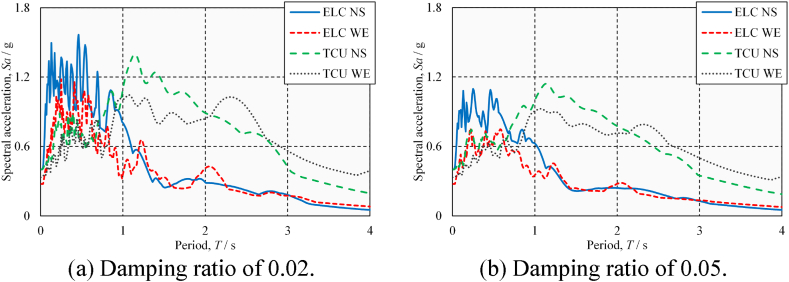
Acceleration response spectrum.

Fig. 8(a) and (b) show the acceleration spectra of a single-degree-of-freedom system with different damping ratios at four horizontal components (amplitude scaling to PGA = 0.4 g) of two ground-motion records (ELC and TCU), respectively. It can be seen that, due to the lower damping ratio of steel bridges, their acceleration responses under short periods (<1 s) are more significant than those of reinforced concrete bridges. The damping ratio of the steel bridge analyzed in this study is low and the period is short; as such, its seismic response and damage are worthy of attention.

## 4 Numeric results

The bridge model was dynamically analyzed and solved via Abaqus. The calculation is divided into three steps: 1) calculate the dynamic properties using the subspace method; 2) consider the gravitational load (static load); 3) consider the result of step two as the initial condition and analysis the seismic load (dynamic load). To obtain better stability and adapt to long-time seismic loads, the calculation is done by the implicit solver.

### 4.1 Dynamic characteristics

**Fig. 9:**
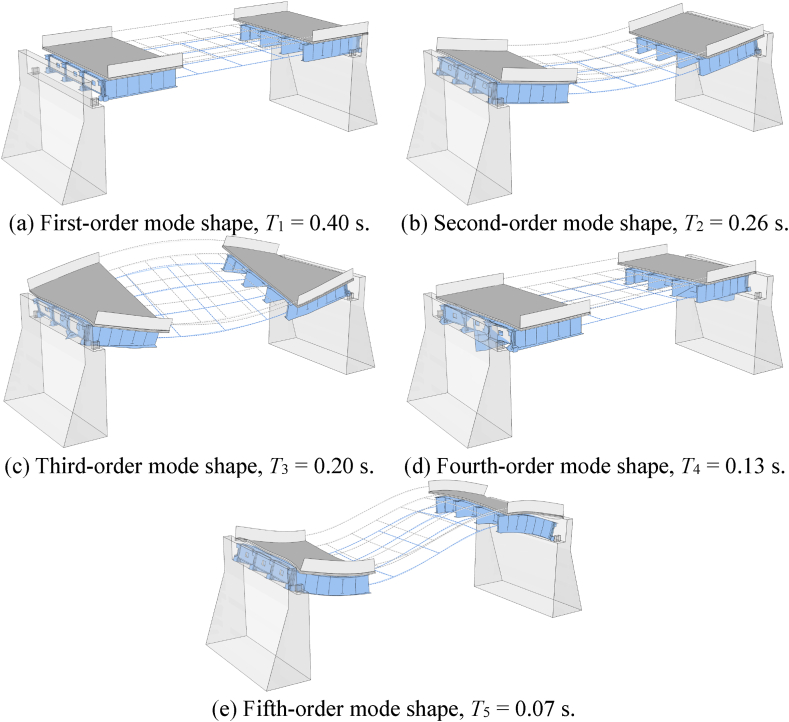
First five-order mode shapes of natural vibration.

Before the time-history analysis, the dynamic characteristics of the bridge model are analyzed. The model's first five-order natural vibration characteristics are shown in Table 3, and the corresponding mode shapes are shown in Fig. 9(a) and (b), 9(c), 9(d), and 9(e). From these results, it can be seen that the bridge model demonstrates the overall torsion in the second- and third-order mode shapes. Due to the large stiffness gap between the steel beam and the bridge deck, it is possible to reveal the combined torsion and horizontal response under seismic. However, the cross-sectional area of steel beams is small, and the local stiffness is low (compared with concrete beams); moreover, the complex and combined seismic response may result in local deformation damage, even buckling failure. These seismic response characteristics of steel bridges are worthy of examination.

**Table 3:** Results of natural vibration characteristics.

NO.	Frequency, *f*/Hz	Period, *T*/s	Mode shape description
1	2.49	0.40	Translate along the bridge
2	3.85	0.26	Vertical bending (C-shape)
3	5.00	0.20	Torsion along the bridge
4	7.69	0.13	Vertical torsion
5	14.00	0.07	Vertical bending (S-shape)

### 4.2 Longitudinal seismic-induced pounding

**Fig. 10:**
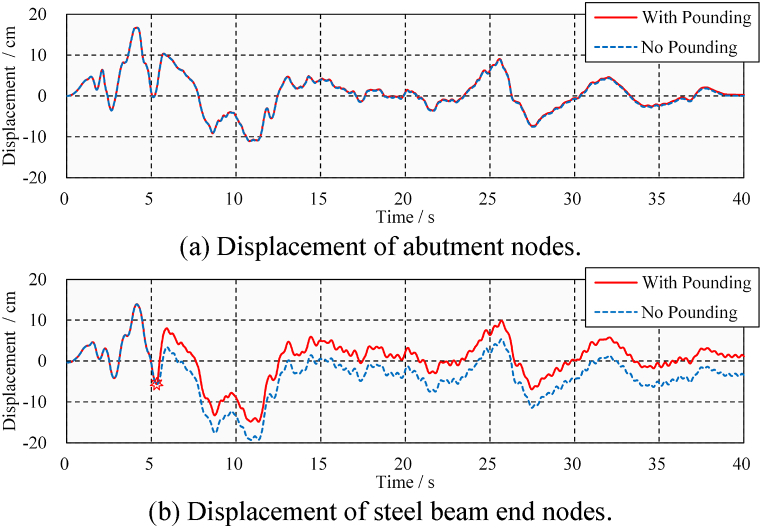
Longitudinal absolute displacement time-history under ELC (PGA = 0.4 g).

In simulating the dynamic response of the bridge under seismic loads, the contact relationship between the abutment and steel beam can be blunted (or activated) to exclude (or consider) the pounding load. Fig. 10 shows the longitudinal absolute displacement response time-history of the bridge abutment and steel beam under ELC ground motion (PGA = 0.4 g). Only the response time-history of the nodes at Gap A Ⅰ is shown for conciseness. As shown in Fig. 10(a), the abutment is almost unaffected by pounding due to its much greater stiffness compared with steel beams. Studies on concrete beams have yielded similar conclusions [32]; that is, the pounding effect on rigid abutments is not evident. However, this pounding event has a great impact on the fragile steel beams. As shown in Fig. 10(b), the steel beam received a single pounding at 5.3 s, resulting in a large impact displacement. However, the maximum relative displacement of the beam decreased by 24.27 % in the whole process. Objectively, Gap A limits the excessive displacement of the bridge, which is beneficial in preventing bearing and beam falling failures. Otherwise, the relative shear displacement of the bearing will exceed the design value of 10 cm, and the risk of falling beam failure is significantly increased. This fact differs from a large number of studies in buildings, which tend to emphasize increasing gaps to avoid seismic-induced pounding [33,34].

**Fig. 11:**
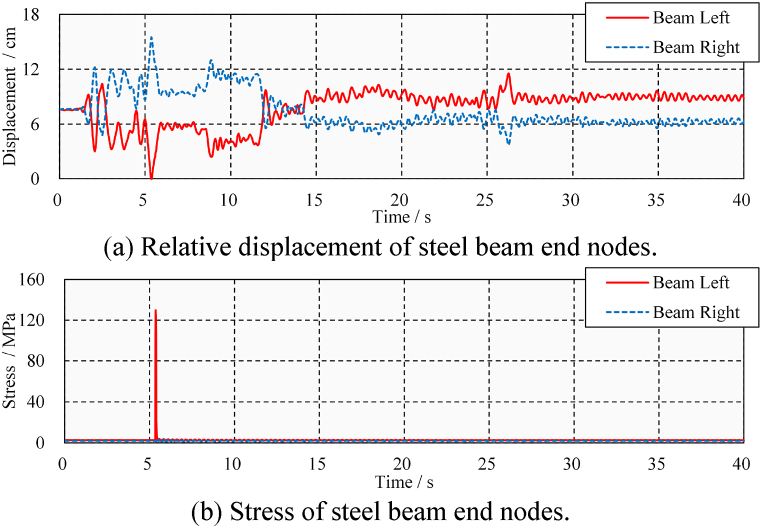
Displacement and stress time-history under ELC (PGA = 0.4 g).

However, it is also necessary to discuss whether seismic pounding due to the presence of a gap will cause damage to the bridge. Fig. 11 shows the displacement and stress time-history of a steel beam considering pounding conditions. Response of a steel beam at positions Ⅰ and Ⅲ is plotted in the figure. It can be seen that the steel beam only had one pounding at the left end. As shown in Fig. 11(a), when the pounding event occurs, the relative distance between the left end of the beam and the abutment drops to zero. Due to the impact force, the stress at the bottom of the steel beam sharply rises to 130 MPa in Fig. 11(b) from a small value at the time of pounding and then drops to a small value. The stress before and after the pounding event is the same, indicating that the steel beam does not encounter residual stress, and its mechanical behavior is within the elastic range. It can be concluded, that under the ELC ground motion (PGA = 0.4 g), only one seismic-induced pounding occurs at the beam's left end. The pounding causes peak stress in the steel beam, but the steel beam is not damaged.

**Fig. 12:**
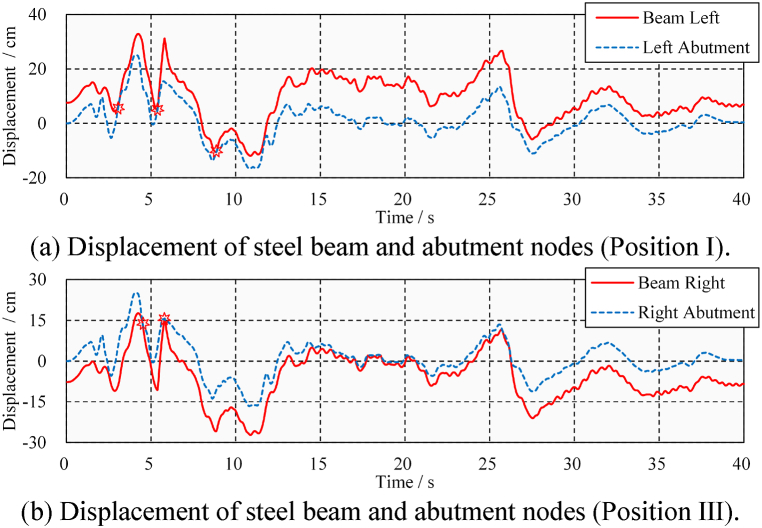
Longitudinal absolute displacement time-history under ELC (PGA = 0.6 g).

Of course, in the event of large ground motion, the bridge's pounding response will be more violent. Fig. 12 shows the longitudinal absolute displacement response time-history of the bridge abutment and steel beam under ELC ground motion (PGA = 0.6 g). It can be seen that both ends of the steel beam (positions I and III) will repeatedly pound with the abutment. For example, as shown in Fig. 12(a), the steel beam first pounds with the left abutment at 5.3 s. Then, as shown in Fig. 12(b), the steel beam immediately pounds with the right abutment at 5.8 s. These two pounding events are only 0.5 s apart, and the short and large pounding force causes the beam's displacement curve to change steeply.

**Fig. 13:**
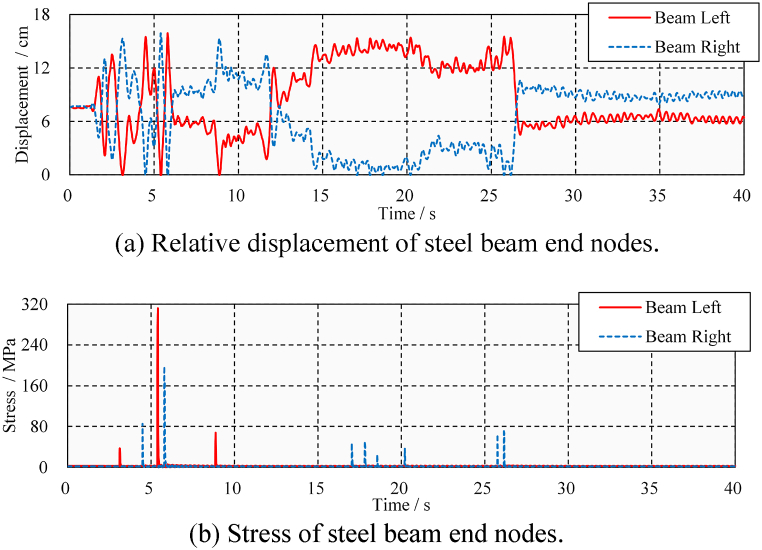
Displacement and stress time-history under ELC (PGA = 0.6 g).

Fig. 13 shows the displacement and stress time-history of the steel beam, which can help us to understand whether these poundings can cause damage. Each pounding of the steel beam and the abutment shown in Fig. 13(a) results in peak stress in Fig. 13(b). It can be seen that the left end of the steel beam has experienced a total of three times poundings, while the right end has experienced eight times poundings. The pounding of 5.3 and 5.8 s caused peak stress of 310 and 200 MPa to the steel beam, respectively. Fortunately, after these poundings, the steel beam is still in an elasticity state. It is concluded that, under the action of ELC ground motion (PGA = 0.6 g), repeated seismic-induced poundings occur in the steel beam but do not cause damage.

**Fig. 14:**
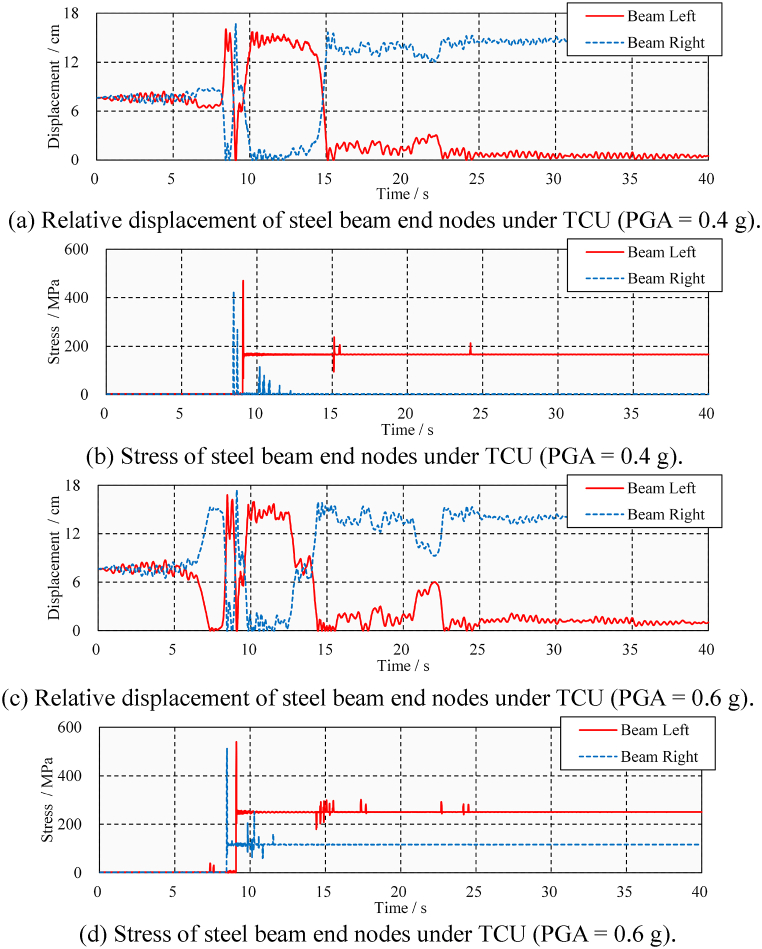
Displacement and stress time-history under TCU.

Near-fault ground motion is generally considered to cause greater pounding damage to the structure due to its characteristic of strong velocity pulses [35,36]. According to Section 3.1, TCU ground motion is selected to discuss the longitudinal pounding damage of steel beams under medium and large ground motions (PGA = 0.4 g and PGA = 0.6 g). As shown in Fig. 14(a), (b), 14(c), and 14(d), the steel beams have more longitudinal poundings and damage under the TCU ground motion. In the case of medium TCU ground motion (PGA = 0.4 g), there were four and eight times poundings at the steel beam's left and right ends, respectively. The peak stress of 460 MPa is significantly higher than the result of ELC (130 MPa). The appearance of residual stress (166 MPa) at the left end of the steel beam indicates that the steel beam has pounding damage. In the case of large TCU ground motion (PGA = 0.6 g), the peak stress and residual stress of the steel beams were increased 540 and 250 MPa, respectively. The damage caused by these poundings to steel beams has been confirmed by the presence of residual stress; however, the extent of the damage needs to be further discussed.

**Fig. 15:**
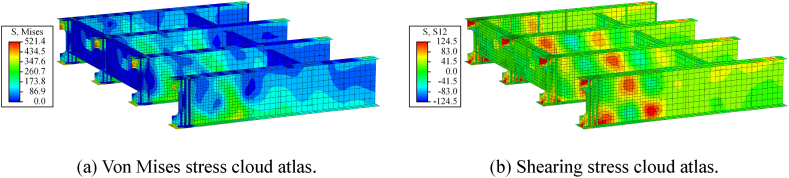
Stress cloud atlas of the steel beams (unit: MPa).

Fig. 15 shows the stress cloud atlas of the steel beams during a pounding event. For ease of observation, an end diaphragm has been omitted in Fig. 15. As shown in Fig. 15(a), the stress and damage caused by the pounding are concentrated at the end of the steel beam. This damage is similar to the seismic damage shown in Fig. 1(a). However, the steel beam remains elastic after the end diaphragms. In addition, as shown in Fig. 15(b), the pounding results in a significant increase in the shear stress at the bottom of the web. This is due to the rigid concrete diaphragm transferring the pounding force from the beam end to the web. The pounding load and gravity load will work together on the steel beam web behind the bearing, thus increasing the risk of shear instability in the web.

In general, the longitudinal seismic-induced pounding shock on steel beams is obvious. The presence of the gap prevents excessive displacement of the beam but also leads to the beam being subjected to pounding loads. All longitudinal pounding records of steel beams are summarized in Table 4. Notably, under large near-fault ground motion, pounding will cause damage to both ends of the steel beam. However, the damage is concentrated at the beam end. The force at the beam end is small under normal use, and the longitudinal stiffness is large; thus, the steel beam will not reduce the bearing capacity due to pounding damage but the risk of shear instability in the web is increased. It is concluded that the longitudinal seismic-induced pounding caused mild damage to the steel beam, and the bridge could be used normally.

**Table 4:** Longitudinal pounding records of steel beams.

Ground Motion	PGA/g	Number of Poundings		Peak Stress/MPa		Residual Stress/MPa	
		Left Ⅰ	Right Ⅲ	Left Ⅰ	Right Ⅲ	Left Ⅰ	Right Ⅲ
ELC (Far-fault)	0.4	1	0	130	0	0	0
	0.6	1	3	310	200	0	0
TCU (Near-fault)	0.4	2	2	460	427	166	0
	0.6	1	3	540	499	250	116

### 4.3 Lateral seismic-induced pounding

**Fig. 16:**
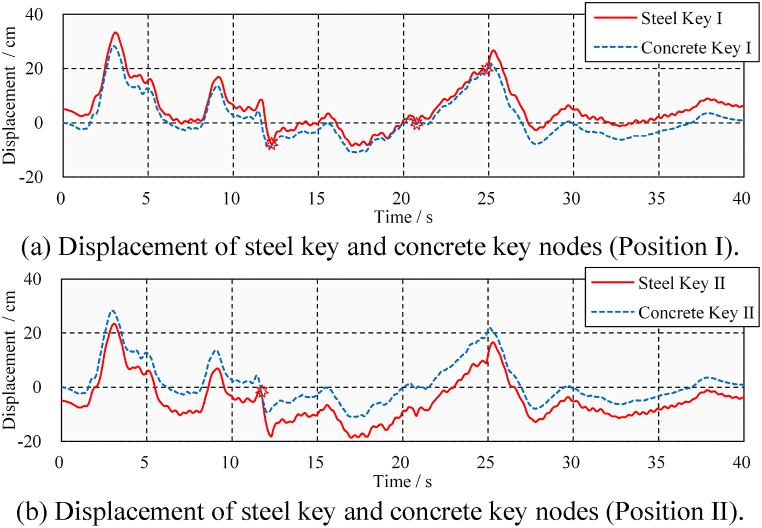
Transverse absolute displacement time-history under ELC (PGA = 0.4 g).

The contact relationship between the concrete keys of the abutment and the steel keys of the steel beams is activated in regard to the phenomenon of lateral seismic-induced pounding. Fig. 16(a) and (b) show the transverse absolute displacement response time-history of concrete keys and steel keys under ELC ground motion (PGA = 0.4 g). Only the response time-history of the nodes at Gap B Ⅰ and Gap B Ⅱ is shown for conciseness. It can be seen that there have been many poundings between the concrete and steel keys. However, due to the presence of Gap B, the maximum relative displacement of the beam decreased by 22.65 % and prevented the bearing failure and lateral beam falling failure.

**Fig. 17:**
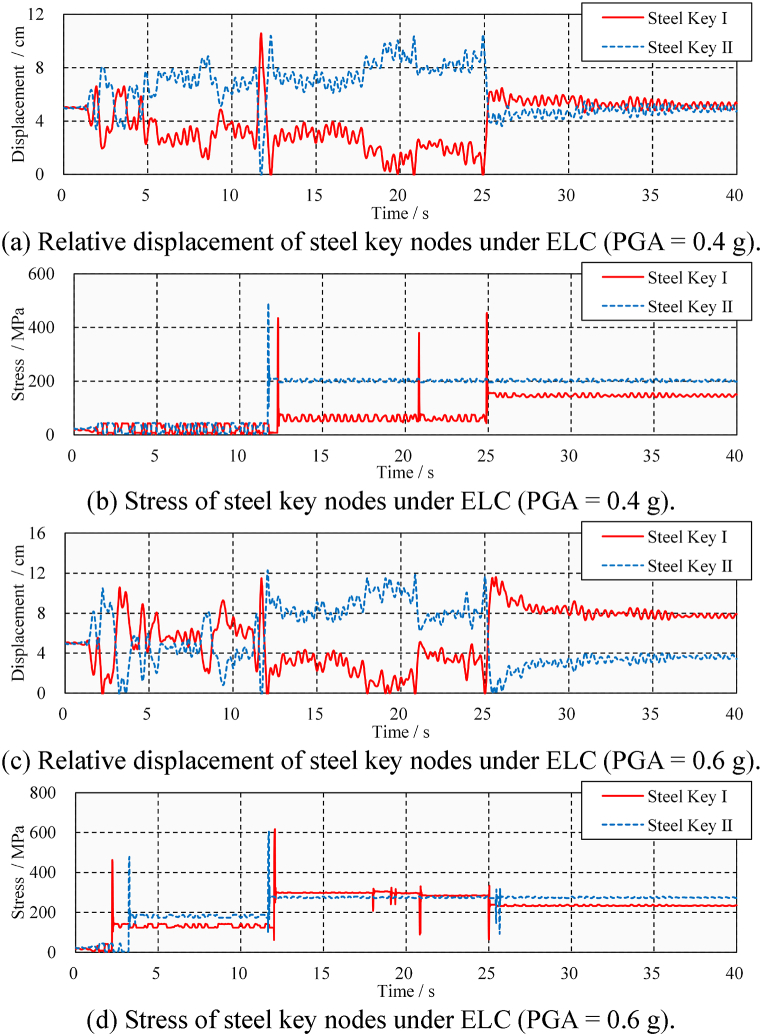
Displacement and stress time-history under ELC.

However, the lateral pounding of the steel keys resulted in significant damage to the steel beams. Fig. 17 shows the displacement and stress time-history at the junction between the steel key and the steel beam under ELC ground motion. As shown in Fig. 17(a), in the case of medium ELC ground motion (PGA = 0.4 g), there were three and one times poundings at the Steel Key I and Steel Key II, respectively. As shown in Fig. 17(b), the peak stress due to the poundings is 491 MPa, and the residual stress is 200 MPa. The presence of residual stress means that the steel beam has undergone plastic deformation and is damaged. Taking Steel Key Ⅰ as an example, it can be found that the residual displacement gradually increases with the number of poundings, indicating that the damage to the steel beam gradually increases. As shown in Fig. 17(c) and (d), the pounding damage is further aggravated under large ELC ground motion (PGA = 0.6 g). There were seven- and four-times poundings at the Steel Key I and Steel Key II, respectively. The peak stress due to the poundings is 611 MPa, and the residual stress is 276 MPa. It is concluded that, under the action of ELC ground motion, repeated seismic-induced poundings occur in the steel beam and cause damage. Compared with the analysis results of longitudinal seismic-induced pounding shown in Section 4.3, it can be found that the lateral pounding damage of steel beams is much more serious even under the far-fault ground motion.

**Fig. 18:**
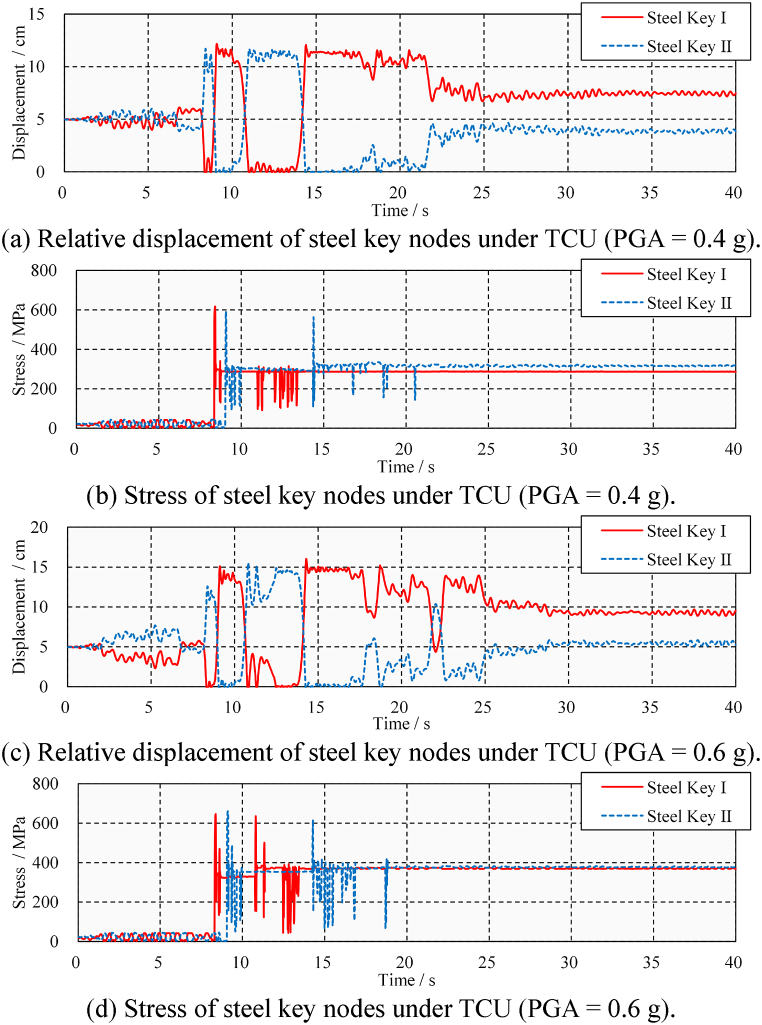
Displacement and stress time-history under TCU.

Fig. 18(a) and (b), 18(c), and 18(d) show the displacement and stress time-history at the junction between the steel key and steel beam under TCU ground motion. It can be seen that the number of pounding events is higher, resulting in greater peak and residual stresses of the steel beam. However, in dense pounding responses, it becomes difficult to identify pounding events based on the displacement time-history. Steel keys may not separate immediately after pounding with concrete keys but are extruded repeatedly. The impact of pounding and extrusion on steel beams is different, i.e., the pounding will lead to residual stress and damage on steel beams, while extrusion only produces an elastic response on the steel beams. Therefore, it is necessary to identify significant pounding events in order to locate and evaluate the damage they cause.

In general seismic time-history analysis, it is difficult and costly to obtain the stress of a structure, while obtaining displacement is simple and convenient. A method that can accurately identify pounding events based only on displacement data is thus proposed. The detailed theory and more cases of this proposed method are shown in Appendix A.

**Fig. 19:**
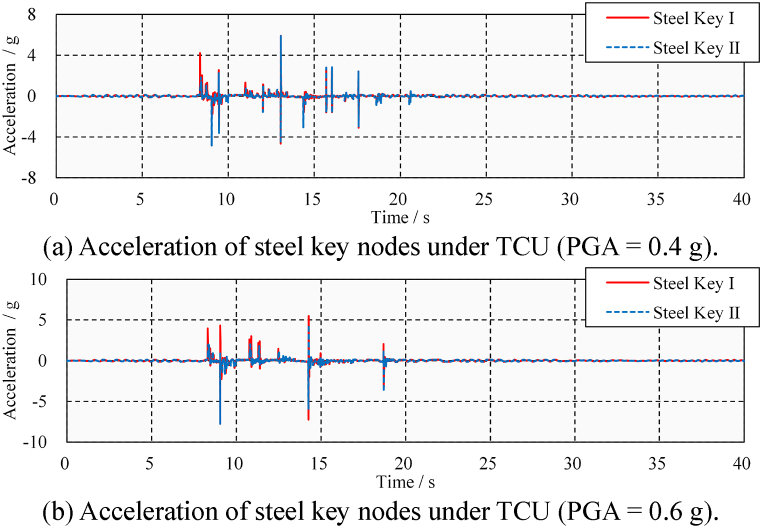
Transverse acceleration time-history under TCU.

First, the acceleration time-history needs to be calculated. The acceleration pulses generated by the poundings propagate in the form of fluctuations to the far end of the beam [37]. The acceleration time-history of the steel keys is shown in Fig. 19(a) and (b). Although poundings cause acceleration peaks, so do extrusions. Pounding events are difficult to identify by acceleration peaks. Moreover, such an identification method is location-dependent. If the pounding occurs in the chosen position rather than on its opposite side, then the pulse is clear. For example, pounding occurs at Steel Key I at 8.4 s, and a large pulse appears in its acceleration time-course. However, since the bridge does not undergo rigid body displacement, Steel Key II on the opposite side of Steel Key I shows only a weak pulse. The opposite situation also occurs at 9.0 s. Selecting only one location for identification may overlook some poundings due to the presence of inconspicuous pulses.

**Fig. 20:**
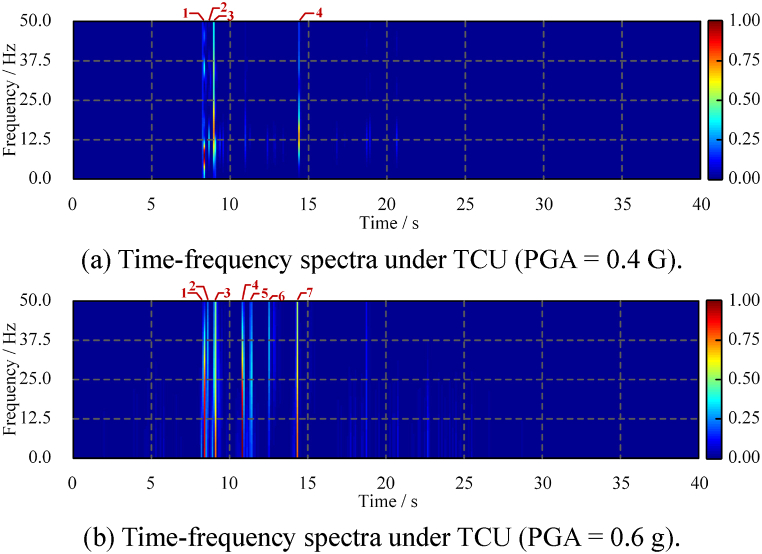
Time-frequency spectra of the transverse acceleration time-history under TCU.

Second, the short-term Fourier transform is performed on the acceleration time-history. The time-frequency analysis result is shown in Fig. 20. Fig. 20(a) and (b) show the analysis results of a steel beam under medium and large TCU ground-motions, respectively. A total of four pounding events are identified in Fig. 20(a), corresponding to times of 8.4, 8.7, 9.1, and 14.4 s. The relative power spectral density shows that the energy from the first and third poundings is significant and causes primary damage. Extrusion events are marked by dotted boxes in the figure, and their relative power density is low and easily distinguishable from poundings. These conclusions are consistent with the stress results shown in Fig. 18(b). In addition, seven poundings are identified in Fig. 20(b), of which the first, third, fourth, and seventh poundings caused more damage. These conclusions are consistent with the stress results shown in Fig. 18(d).

**Fig. 21:**
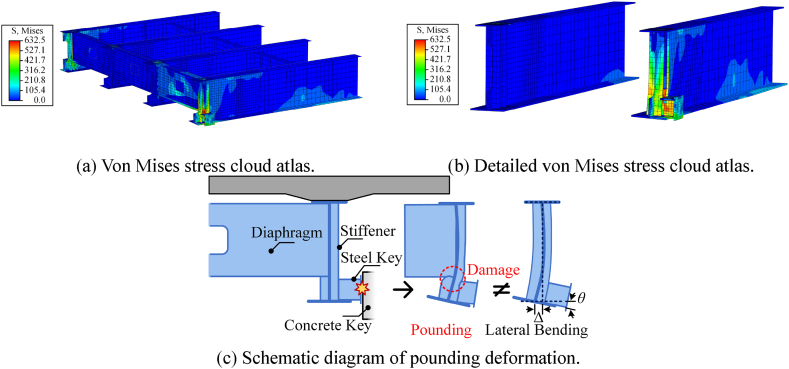
Stress and deformation of the steel beams (unit: MPa).

Lateral poundings of steel beams are prone to occur and cause higher stresses. Therefore, the damage to steel beams needs to be further discussed. As shown in Fig. 21(a), the stress and damage caused by the pounding are concentrated in the middle and lower parts of the steel beam. However, due to repeated poundings on the side steel beams, the damaged area includes the steel web, beam ends, stiffeners, diaphragms, and steel keys. In addition, as shown in Fig. 21(b), the steel beam will undergo significant lateral deformation. This damage is similar to the seismic damage shown in Fig. 1(b). The deformation is irreversible because the damaged area enters a plastic state. Fig. 21(c) is the schematic diagram of pounding deformation. The lateral bending deformation is also presented in Fig. 21(c), unlike lateral bending [38] the pounding not only causes the rotation and displacement of the beam bottom but also causes irreversible damage. The damaged areas are located directly above the bearing, where the welds meet and the forces are complex. In addition, the second-order effect of gravity due to the lateral bending of steel beams has a more adverse effect. It can be seen that the damage to steel beams caused by lateral seismic-induced poundings is much more serious than that caused by longitudinal poundings. The main reason why lateral pounding damage is so severe is due to the uneven lateral stiffness of the steel beam. The deformation is concentrated in the stiffness mutation zone after the steel key is pounded. The arrangement of a rigid diaphragm and vertically staggered steel key is not conducive to the transmission of pounding force.

**Fig. 22:**
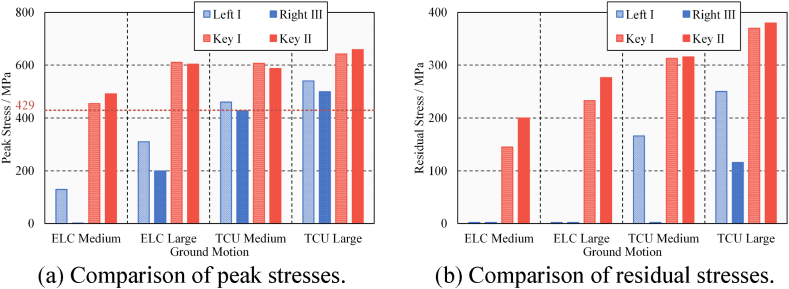
Longitudinal and lateral pounding damage of the steel beam.

In general, the lateral seismic-induced pounding shock on steel beams is significant. The presence of shear keys prevents the lateral beam falling failure but also leads to possible lateral pounding damage to the steel beam. All lateral pounding records of steel beams are summarized in Table 5. The results of peak stress and residual stress due to longitudinal and lateral poundings are compared in Fig. 22(a) and (b). Longitudinal results are represented by Left I and Right III; horizontal results are represented by Key I and Key II. The red grid line indicates that the steel's yield strength is 429 MPa. Once the peak stress exceeds the yield strength, it means that the steel beam enters the plastic state, and there is residual stress and damage. Lateral poundings cause more serious damage in each case of ground motion. The near-fault ground motion aggravated the pounding damage on the steel beam. Moreover, the damage is concentrated in areas of high and complex stress. Comprehensively considering these situations, the steel beam may reduce the bearing capacity due to pounding damage. It is concluded that the lateral seismic-induced pounding caused moderate to severe damage to the steel beam, so whether the bridge could be used normally needed to be evaluated.

**Table 5:** Lateral pounding records of steel beams.

Ground Motion	PGA/g	Number of Poundings		Peak Stress/MPa		Residual Stress/MPa	
		Key Ⅰ	Key Ⅱ	Key Ⅰ	Key Ⅱ	Key Ⅰ	Key Ⅱ
ELC (Far-fault)	0.4	3	1	454	491	145	200
	0.6	3	2	611	604	233	276
TCU (Near-fault)	0.4	2	2	607	587	313	316
	0.6	5	2	643	659	370	380

### 4.4 Horizontal seismic-induced pounding

**Fig. 23:**
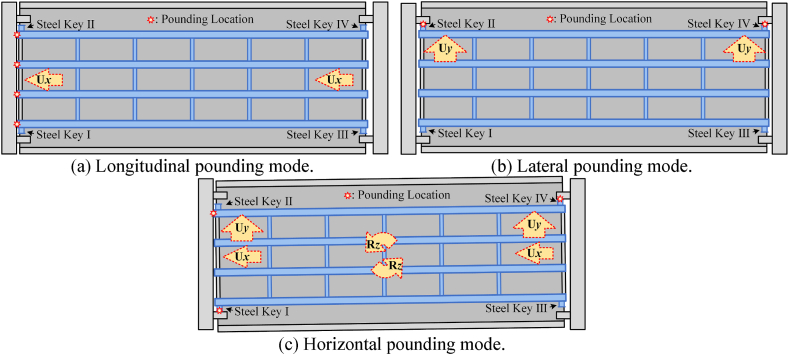
Pounding modes of the seismic-induced steel bridge.

Under separate longitudinal or transverse ground-motion components, the steel bridge will experience longitudinal or lateral seismic-induced pounding. Ground motion in the horizontal direction consists of two components acting simultaneously. Fig. 23 shows the pounding modes of the steel bridge. As shown in Fig. 23(a), the longitudinal pounding mode represents the pounding between the steel beam ends and the abutment. As analyzed in Section 4.2, the pounding force is carried by all steel beams and results in limited damage to the steel beam ends. As shown in Fig. 23(b), the lateral pounding mode represents the pounding between the steel keys and the concrete keys. As analyzed in Section 4.3, the pounding force is only carried by both sides of steel beams and results in significant damage to the steel beams. When the two components work together, the horizontal ground motion not only changes in magnitude but also in direction with time. The change in the direction of ground motion causes the vertical rotation of the bridge [39]. Due to such rotation, the large pounding force cannot be evenly carried by steel beams but concentrated to corner points. As shown in Fig. 23(c), the horizontal pounding mode represents the pounding at the steel beam ends and steel stops; further, the pounding force will be carried by a few parts. Thus, the contact relationships in sections 4, 4.2.3 are activated simultaneously to account for the phenomenon of horizontal seismic-induced pounding.

**Fig. 24:**
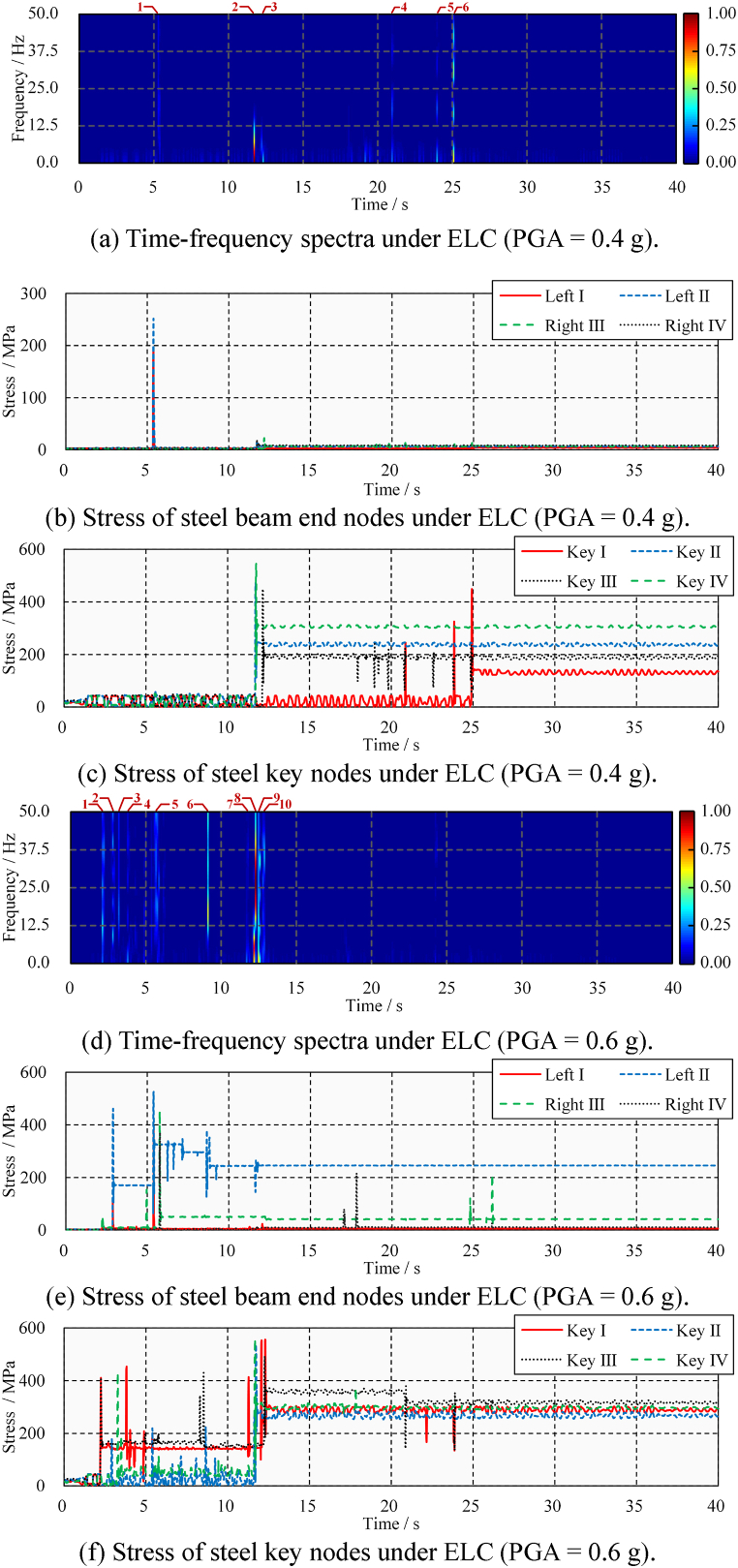
Time-frequency spectra and stress time-history and under ELC.

Fig. 24 shows the time-frequency spectra and stress time-history of steel beams under ELC ground motion. The identification of all pounding events under medium ground-motion is shown in Fig. 24(a), revealing that a total of six pounding events occurred, and the impact of the second (at 11.7 s) and sixth (at 24.9 s) was large. This basic understanding is confirmed by the stress time-history. As shown in Fig. 24(b), the steel beams’ ends remain elastic and undamaged. However, as shown in Fig. 24(c), all the steel beams connected to the steel keys have been damaged. Due to the rotation of the steel beam under the horizontal ground-motion, the damage process and degree of the four steel keys are different. A comparison with Fig. 17(b) shows that the lateral pounding damage is more severe, especially for Steel Key Ⅳ. The peak and residual stress are increased by 11.2 % and 55.0 %, respectively. The identification of all pounding events under large ground motion is shown in Fig. 24(d), revealing that a total of 10-times pounding events occurred, and the impact of the sixth (at 8.6 s), eighth (at 11.6 s), and ninth (at 12.6 s) was large. As shown in Fig. 24(e) and (f), pounding damage occurred to the beam ends and steel keys. A comparison with Fig. 17(b) shows that the lateral pounding damage is more severe, especially for Steel Key Ⅲ.

**Fig. 25:**
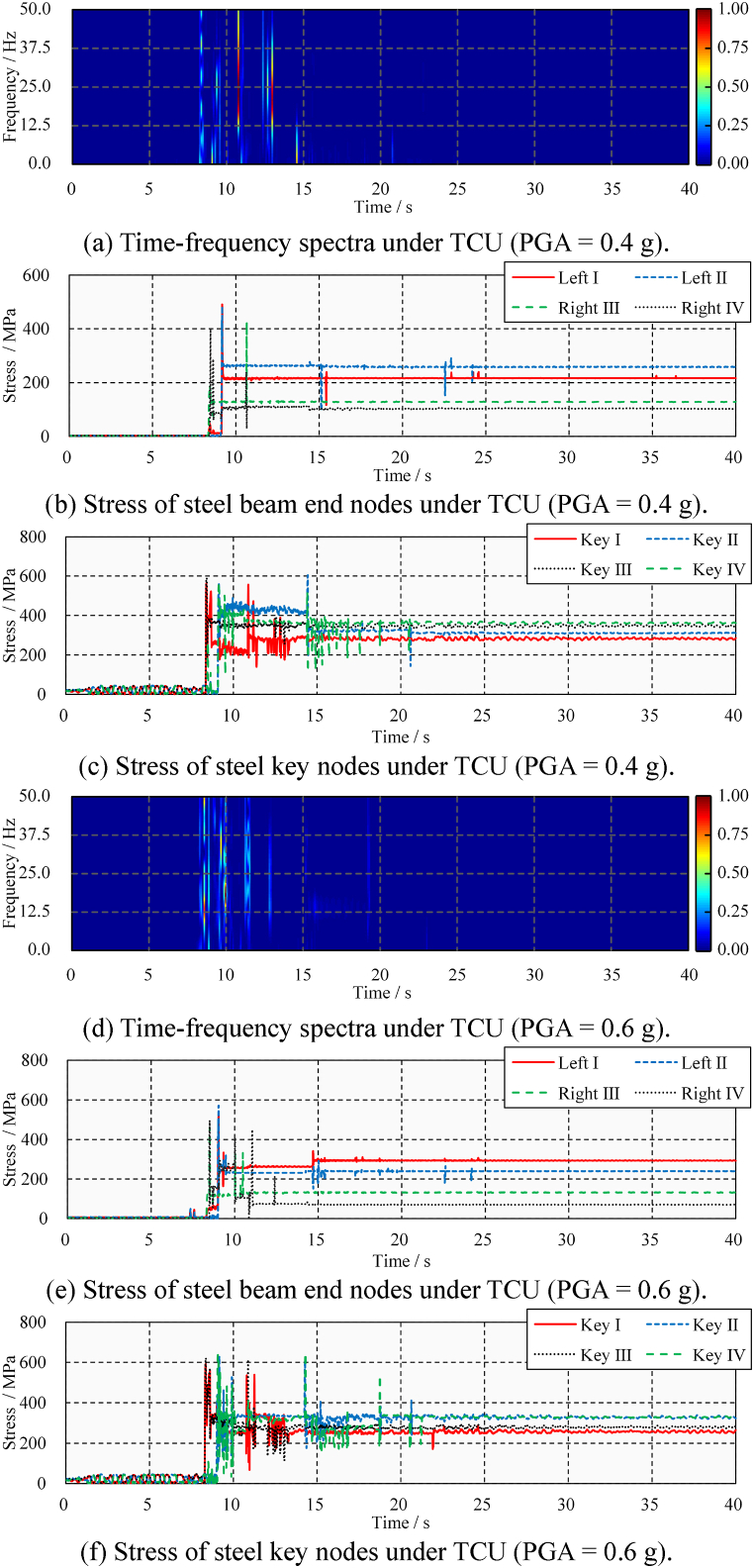
Stress time-history and time-frequency spectra under TCU.

Fig. 25(a) and (b), 25(c), 25(d), 25(e), and 25(f) show the time-frequency spectra and stress time-history of steel beams under TCU ground-motion. It can be found that the damage to the steel beam is obvious even under medium ground-motion. Near-fault ground motion can lead to more severe damage to the steel bridge. A comparison with Fig. 18 shows that the lateral pounding damage of steel beams under horizontal ground-motion is more serious. This is due to uneven pounding caused by the in-plane rotation of the bridge. For example, at 10.9 s in Fig. 25(c), pounding is concentrated in Steel Key I.

**Fig. 26:**
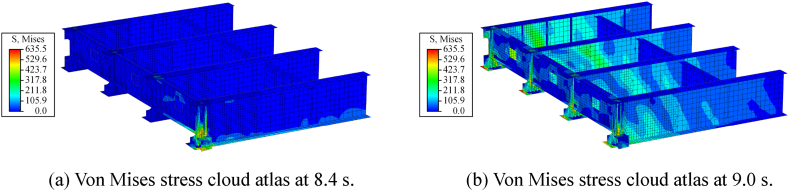
Stress cloud atlas of the steel beams (unit: MPa).

The details of the uneven pounding of steel beams caused by horizontal ground-motion are shown in Fig. 26. At 8.4 s, the lateral pounding is concentrated on Steel Key I. After 0.6 s, the steel beam immediately underwent longitudinal pounding on Beam End II. The pounding damage situation of the steel key and beam end is different. As shown in Fig. 26(a), the pounding of the steel key will cause damage to the lower half of the steel beam. The further damaged area includes the steel web, beam ends, stiffeners, diaphragms, and steel keys. The damage to the steel beam's end is limited before the end diaphragm, as shown in Fig. 26(b). It is concluded that damage from lateral pounding remained the main one.

**Fig. 27:**
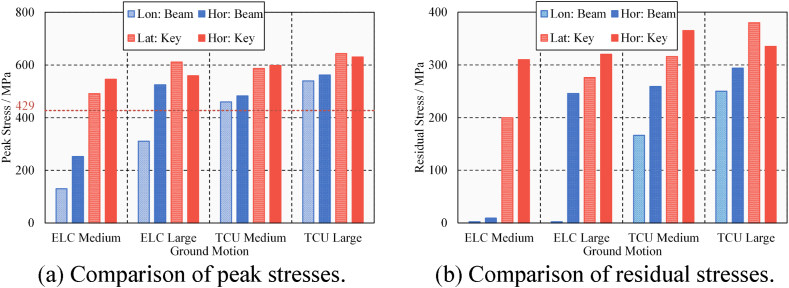
Longitudinal and lateral pounding damage of the steel beam.

The results of maximum peak stress and residual stress due to longitudinal, lateral, and horizontal poundings are compared in Fig. 27(a) and (b). For longitudinal poundings, only the results of the beam ends are considered (represented by Lon: Beam); for lateral poundings, only the results of the keys are considered (represented by Lat: Key); and for horizontal poundings, the results of beam ends and keys are considered (represented by Hor: Beam and Lat: Key, respectively). The steel's yield strength is 429 MPa. Once the peak stress exceeds the yield strength, the steel beam enters the plastic state, and there is residual stress and damage. In general, the damage from horizontal poundings is obvious. The results are more serious due to large and near-fault ground motions. The peak and residual stresses have increased significantly under horizontal ground motions, considering the scope and degree of damage, the lateral pounding of the keys still mainly caused damage to the steel beams. The pounding concentration caused by the rotation of the bridge deck notably deepens the damage to the steel beams. It is concluded that the lateral damage of steel beams is aggravated by the combined effect of transverse and longitudinal ground-motions. Therefore, it is recommended to perform horizontal two-way ground-motion analysis in seismic design to avoid underestimating pounding damage.

### 4.5 Prevention measure

**Fig. 28:**
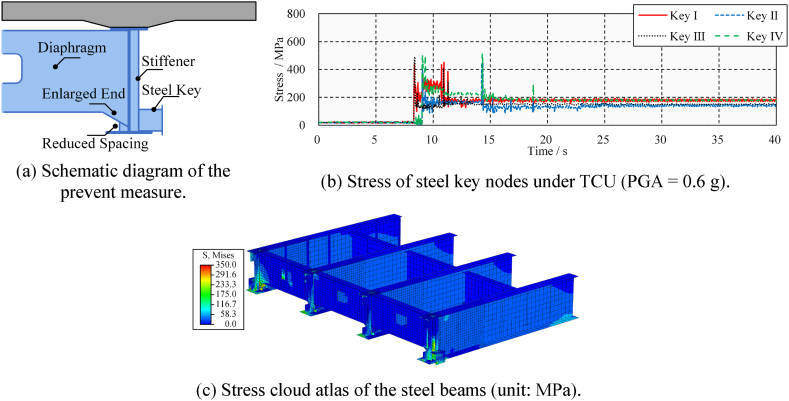
Measure to prevent pounding damage.

According to the numerical results, the seismic-induced pounding damage mainly occurs in the lateral direction of the side beam. Due to the end diaphragm, there is a mutation in the overall transverse stiffness of the steel beam end. When the steel key is carried to the lateral pounding force, the horizontal and rotational displacement is concentrated at the bottom plate; thus, there is a significant stress concentration in the connection area between the displacement and the steel beam. To avoid the bending-shear failure of the steel beam due to the lateral pounding, it should be ensured that the beam's lateral stiffness is gradually transitioned. A feasible preventive measure is shown in Fig. 28(a). Key points of this measure include an enlarged diaphragm end and reduced beam bottom space. The enlarged diaphragm is conducive to the gradual transition of transverse stiffness, and the reduced beam bottom space is conducive to the diffusion of pounding force.

To verify the effectiveness of this preventive measure, the response of the modified bridge model under TCU ground-motion (PGA = 0.6g) is calculated. The stress time-history of the four steel keys is shown in Fig. 28(b). It can be seen that the peak and residual stress are significantly decreased compared to Fig. 25(f). The average peak and residual stress of Steel Keys I to IV decreased by 23.3 % and 44.8 %, respectively. The cloud atlas of the steel beams’ pounding damage is shown in Fig. 28(c). The lateral deformation of the steel beams is controlled due to the providing support of enlarged diaphragm ends. Moreover, the pounding force is dispersed and borne by the diaphragm, which significantly reduces the plastic damage of steel beams. It can be seen that the proposed measures prevent serious lateral pounding damage in steel bridges.

## 5 Conclusions

In the present paper, the pounding damage of seismic-induced steel bridge was studied in detail. The multiscale fine numerical model of a steel–concrete composite girder bridge was established and nonlinear behaviors of a steel beam and bearing were considered. The three-dimensional arbitrary contact relationship for components that may experience poundings was established. This contact scheme does not require preset pounding positions, so any three-dimensional poundings of point-to-surface and surface-to-surface can be accurately simulated. The finite element calculation platform Abaqus was used to investigate the pounding response and damage of steel bridge under far-fault and near-fault ground motions in detail. The following conclusions can be drawn from this research.

(1) According to the steel bridge's dynamic characteristics, the stiffness gap between the steel beam and the bridge deck is large. The steel beam's cross-sectional area is small, and its local stiffness is low, the seismic response may result in local deformation damage.
(2) Longitudinal pounding causes damage to the steel beam's ends. The area and grade of damage are localized and mild; however, pounding also increases the risk of shear instability in the web.
(3) Lateral pounding causes direct damage to the steel beams. The area and grade of the damage are extensive and may reduce the steel beam's bearing capacity. In any case of ground motion, especially the near-fault one, lateral poundings cause more damage than longitudinal poundings. In addition, the pounding damage range and deformation mechanism are clarified.
(4) Horizontal two-way pounding causes rotation of the bridge deck, which brings unevenness and concentration of pounding. The lateral damage of steel beams is aggravated by the combined effect of transverse and longitudinal ground motions. The seismic pounding modes are summarized and it is recommended to perform horizontal two-way ground motion analysis in seismic design to avoid underestimating the pounding damage.
(5) A feasible preventive measure for lateral pounding damage is suggested based on the mechanical characteristics of pounding damage, which solves the design defects. The identification method based only on displacement data is proposed, which can easily and accurately identify all poundings that occur in steel beams.

## Data availability statement

No data was used for the research described in the article.

## Ethics declaration

Review and/or approval by an ethics committee was not needed for this study because the object of this study is engineering structures.

## CRediT authorship contribution statement

**Fan Shi:** Writing – original draft, Methodology. **Dongsheng Wang:** Writing – review & editing, Funding acquisition. **Lei Tong:** Validation. **Weijian Tang:** Visualization.

## Declaration of competing interest

The authors declare that they have no known competing financial interests or personal relationships that could have appeared to influence the work reported in this paper.

## Figures and Tables

**Fig. A1:**
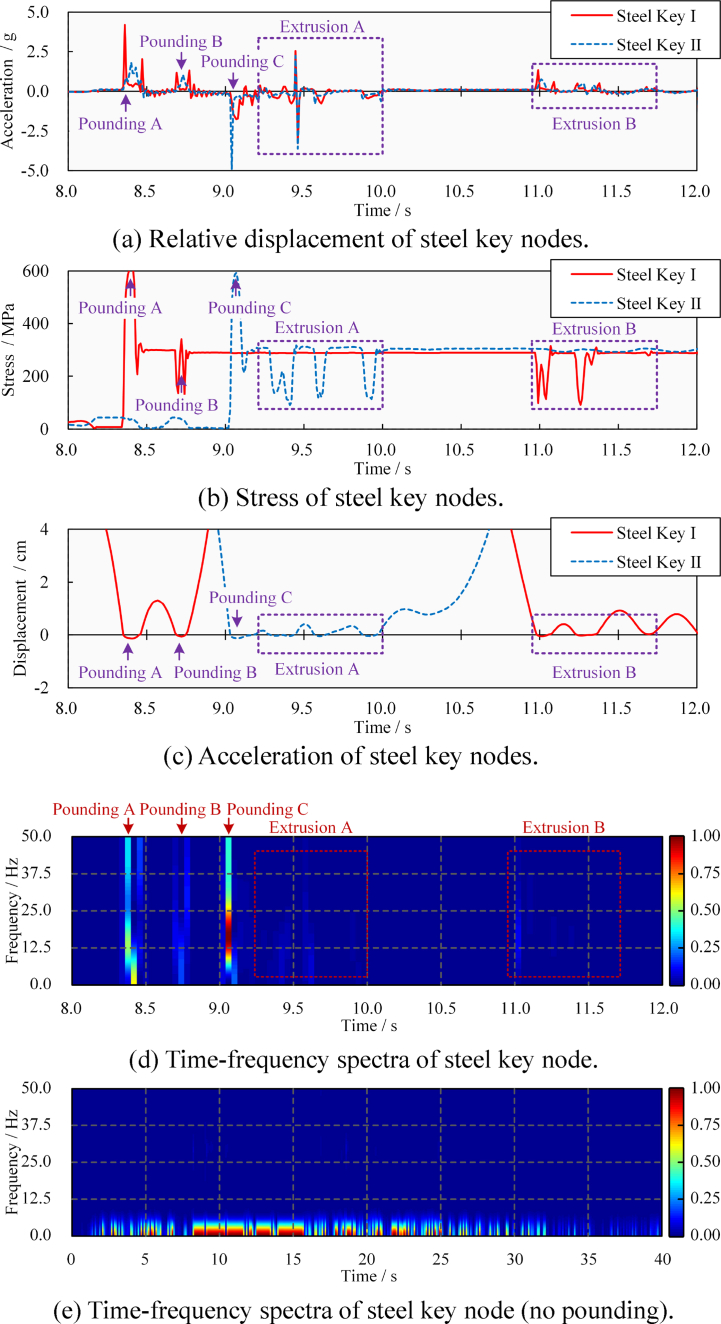
Case of the pounding identification method.

## Appendix A Identification method for pounding events

The motion behavior of steel bridges is complex during ground-motions and often includes pounding responses. Although seismic-induced pounding can be obtained through detailed numerical analysis, it is difficult and costly to obtain the stress time-history in practice. In Section 4.3, in order to accurately identify the lateral seismic-induced poundings of steel beams, an identification method based only on displacement data is proposed. The detailed theory and more cases of the proposed method are given in this Appendix.

As shown in Fig. A1(a), both pounding events and extrusion events occur between the steel and concrete keys. The significant difference between pounding and extrusion is whether it acts in a short period of time and then separates quickly. From a stress perspective, as shown in Fig. A1(b), pounding results in peak stress and residual stress, which are not results of extrusion. A typical pounding load acting on a beam causes an instantaneous directional change in its displacement. The velocity of the beam will also undergo a process of momentary decline and growth. A large pounding load must have a large pulse in the steel beam's acceleration time-history [40]. Therefore, a direct consideration for identifying pounding events is to obtain the acceleration time-history of the steel beam. The acceleration time-history can be obtained using the differential method from the displacement time-history. The second-order forward difference method is adopted and calculated as shown in Eq. (A1).

(A1) $$\Delta^{2}dis\left(t_{k}\right)=dis\left(t_{k+2}\right)-2dis\left(t_{k+1}\right)+dis\left(t_{k}\right)\text{,}$$

where Δ is the difference operator, *dis*(−) is the displacement time range, *t* is the time, and *k* is the time-series.

As shown in Fig. A1(c), a pounding event does mean an acceleration peak. However, the extrusion event also introduces an acceleration peak. For example, there is a large acceleration peak in Extrusion A. In addition, this identification method is location-dependent. Steel Key Ⅰ withstands Pounding A, and its acceleration peak is obvious, but Steel Key Ⅱ only has a weak acceleration peak. Steel Key Ⅱ withstands Pounding C, and its acceleration peak is obvious, but Steel Key Ⅰ only has a weak acceleration peak. If the degree of collision is judged only by the peak of acceleration, some extrusion events that do not cause damage will be incorrectly considered, and the degree of pounding in other locations will be incorrectly reduced.

To distinguish the acceleration caused by pounding and extrusion, time-frequency analysis methods were introduced. The short-time Fourier transform is a method used to analyze time and frequency signals. It is generated by a Fourier transform of a short portion of the signal sampled by the moving window as Eqs. (A2), (A3) [41].

(A2) $$S_{t}\left(\omega \right)=\frac{1}{\sqrt{2\pi }}\int_{-\infty }^{\infty }s_{h}\left(\tau \right)e^{-j\omega \tau }d\tau \text{,}$$

(A3) $$s_{h}\left(\tau \right)=s\left(\tau \right)h\left(\tau -t\right)\text{,}$$

where *ω* is the angular frequency *ω* = 2*πf*, and *h*(*τ*−*t*) is a window function that emphasizes the signal around the time *t* and is a function of *τ*−*t*. For discrete time series, the power spectrum is calculated from Eq. (A4).

(A4) $$STFT\left\{acc\left(t\right)\right\}\left(m,\omega \right)\equiv X\left(m,\omega \right)=\sum_{n=-\infty }^{\infty }acc\left[n\right]win\left[n-m\right]e^{-j\omega n}\text{,}$$

where *acc*[*n*]*win*[*n*] is a stationary signal within different finite time windows, and *m* is the window start time. For the acceleration time-history, we use the Hamming window of length given by Eq. (A5), which has the advantages of relatively wide analytical bandwidth and small energy leakage [42].

(A5) $$win\left[n\right]=\left\{ \begin{array}{cc} 0.54-0.46\cos\left(\frac{2\pi n}{M-1}\right), & 0\leq n\leq M-1\\ 0, & \text{ELSE} \end{array}\right.\text{,}$$

where *n* = 1, 2, …, *N*−1, *N* is the total length of the window function, and *M* is the effective length of the window function.

The time-frequency analysis result of an acceleration record, as shown in Fig. A1(c) using the short-time Fourier transform, is shown in Fig. A1(d). It can be seen that three pounding events are accurately identified, and the extrusion events are almost blanked. The relative power spectral density shows that the energy from Pounding A and Pounding B is significant and causes primary damage. Extrusion A and Extrusion B are marked by dotted boxes in the figure, and their relative power density is low and easily distinguishable from poundings. Identification cases under horizontal bidirectional ground motions are given in Section 4.4. In addition, a situation of no pounding is identified. From Fig. A1(e), it can be seen that the relative power density is evenly distributed, and there is no high-frequency component. Based on the displacement time-history of only one position, all poundings that occur in the steel beam can be identified by the proposed method.

## References

[1] Ritchie P., Kauhl N., Kulicki J. (1999).

[2] Jamshidi M., Majid T A., Bunnori N M., Darvishi A. (2012). Review on seismic behavior of slab-on-girder steel highway bridges. J. Appl. Sci..

[3] Housner G.W., Thiel C.C. (2019). The continuing challenge: report on the performance of state bridges in the Northridge earthquake. Earthq. Spectra.

[4] Kawashima K. (1997). The damage of highway bridges in the 1995 Hyogo-Ken Nanbu earthquake and its impact on Japanese seismic design. J. Earthq. Eng..

[5] Buckle I., Itani A., Carden L. (2010). Recent developments in the seismic design of bridges with steel-plate girder superstructures. J. Earthq. Eng..

[6] Usami T., Ge H. (2011). A performance-based seismic design methodology for steel bridge systems. Journal of Earthquake and Tsunami.

[7] Association J.B. (2016).

[8] Mya Nan A., Kasai A., Shigeishi M. (2018). An investigation of damage mechanism induced by earthquake in a plate girder bridge based on seismic response analysis: case study of Tawarayama bridge under the 2016 Kumamoto earthquake. Adv. Civ. Eng..

[9] Gibe H.A., Tamai H., Sonoda Y. (2020). Numerical study on failure process and ultimate state of steel bearing under combined load. Heliyon.

[10] Mustafa S., Miki C. (2020). Design of rupture strength of side blocks in elevated steel girder bridges with elastomeric bearings. International Journal of Steel Structures.

[11] Jankowski R., Wilde K., Fujino Y. (2000). Reduction of pounding effects in elevated bridges during earthquakes. Earthq. Eng. Struct. Dynam..

[12] Zhu P., Abe M., Fujino Y. (2004). Evaluation of pounding countermeasures and serviceability of elevated bridges during seismic excitation using 3D modeling. Earthq. Eng. Struct. Dynam..

[13] Chouw N., Hao H. (2005). Study of SSI and non-uniform ground motion effect on pounding between bridge girders. Soil Dynam. Earthq. Eng..

[14] Bi K., Hao H., Chouw N. (2009).

[15] Dimitrakopoulos E.G. (2011). Seismic response analysis of skew bridges with pounding deck-abutment joints. Eng. Struct..

[16] Banerjee A., Chanda A., Das R. (2017). Seismic analysis of a curved bridge considering deck-abutment pounding interaction: an analytical investigation on the post-impact response. Earthq. Eng. Struct. Dynam..

[17] Bi K.M., Hao H. (2013). Numerical simulation of pounding damage to bridge structures under spatially varying ground motions. Eng. Struct..

[18] Hao H., Bi K.M., Chouw N., Ren W.X. (2013). State-of-the-Art review on seismic induced pounding response of bridge structures. Journal of Earthquake and Tsunami.

[19] Sha B., Tao T.Y., Xing C.X., Wang H., Li A.Q. (2020). Pounding analysis of isolated girder bridge under nonpulse and pulse-like earthquakes. J. Perform. Constr. Facil..

[20] Li N.N., Xu W.B., Chen Y.J., Yan W.M. (2019).

[21] Xu L.Q., Fu P.Y., Spencer B.F. (2020). Maintaining bridge alignment during seismic events: shear key design and implementation guidelines. J. Bridge Eng..

[22] Meng D.L., Chen S.Z., Yang M.G., Hu S.T. (2021). Effects of shear keys and track system on the behavior of simply-supported bridges for high-speed trains subjected to transverse earthquake excitations. Adv. Struct. Eng..

[23] DesRoches R., Choi E., Leon R.T., Dyke S.J., Aschheim M. (2004). Seismic response of multiple span steel bridges in central and southeastern United States. I: as built. J. Bridge Eng..

[24] DesRoches R., Choi E., Leon R.T., Pfeifer T.A. (2004). Seismic response of multiple span steel bridges in central and southeastern United States. II: retrofitted. J. Bridge Eng..

[25] Won J.H., Mha H.S., Kim S.H. (2015). Effects of the earthquake-induced pounding upon pier motions in the multi-span simply supported steel girder bridge. Eng. Struct..

[26] Zheng Y., Xiao X., Zhi L.H., Wang G.B. (2015). Evaluation on impact interaction between abutment and steel girder subjected to nonuniform seismic excitation. Shock Vib..

[27] Dassault S. (2016).

[28] Shi F., Wang D.S., Chen L. (2023). Cyclic elastoplastic constitutive model for stainless steels compatible with multiple strengths. J. Constr. Steel Res..

[29] Xing Y., Xu Y.N., Guo Q., Jiao J.F., Chen Q.W. (2021). Experimental study on friction performance of damaged interface in steel-concrete composite beam connected by high-strength bolt. Adv. Struct. Eng..

[30] Baker J.W. (2007). Quantitative classification of near-fault ground motions using wavelet analysis. Bull. Seismol. Soc. Am..

[31] Chopra A.K., McKenna F. (2016). Modeling viscous damping in nonlinear response history analysis of buildings for earthquake excitation. Earthq. Eng. Struct. Dynam..

[32] Bi K., Hao H., Chouw N. (2016). 3D FEM analysis of pounding response of bridge structures at a canyon site to spatially varying ground motions. Adv. Struct. Eng..

[33] Abdel Raheem S.E. (2014). Mitigation measures for earthquake induced pounding effects on seismic performance of adjacent buildings. Bull. Earthq. Eng..

[34] Kazemi F., Miari M., Jankowski R. (2020). Investigating the effects of structural pounding on the seismic performance of adjacent RC and steel MRFs. Bull. Earthq. Eng..

[35] Dai K.S., Luo X., Lu Y., Li B., Zhong J., Zhang S.M. (2020). Seismic collision potential of adjacent base-isolated buildings with corridor bridges subjected to bidirectional near-fault pulse-like ground motions. Soil Dynam. Earthq. Eng..

[36] Lin Y.Z., Zong Z.H., Bi K.M., Hao H., Lin J., Chen Y.Y. (2020). Numerical study of the seismic performance and damage mitigation of steel-concrete composite rigid-frame bridge subjected to across-fault ground motions. Bull. Earthq. Eng..

[37] Malhotra P.K. (1998). Dynamics of seismic pounding at expansion joints of concrete bridges. J. Eng. Mech..

[38] Peralta L., Hube M.A. (2018). Deck rotation of straight bridges induced by asymmetric characteristics and effect of transverse diaphragms. Eng. Struct..

[39] Guo A.X., Li Z.J., Li H. (2011). Point-to-Surface pounding of highway bridges with deck rotation subjected to Bi-directional earthquake excitations. J. Earthq. Eng..

[40] He L.-X., Shrestha B., Hao H., Bi K.-M., Ren W.-X. (2016). Experimental and three-dimensional finite element method studies on pounding responses of bridge structures subjected to spatially varying ground motions. Adv. Struct. Eng..

[41] Nagarajaiah S. (2009). Adaptive passive, semiactive, smart tuned mass dampers: identification and control using empirical mode decomposition, hilbert transform, and short-term fourier transform. Struct. Control Health Monit..

[42] Yan Y., Cui Y.F., Guo J., Hu S., Wang Z., Yin S.Y. (2020). Landslide reconstruction using seismic signal characteristics and numerical simulations: case study of the 2017 "6.24" Xinmo landslide. Eng. Geol..

